# Mealtime Care for People With Dementia: What Do Nursing Home Staff Think?

**DOI:** 10.1093/geroni/igaa057.591

**Published:** 2020-12-16

**Authors:** James Faraday, Clare Abley, Catherine Exley, Joanne Patterson

**Affiliations:** 1 Newcastle University, UK, Newcastle upon Tyne, United Kingdom; 2 The Newcastle upon Tyne Hospitals NHS Foundation Trust, UK, Newcastle upon Tyne, United Kingdom; 3 The University of Liverpool, UK, Liverpool, United Kingdom

## Abstract

More and more people with dementia are living in nursing homes (NH). Often, they depend on NH staff for help with eating and drinking. It is important that staff have the skills and support they need to provide good care at mealtimes. This qualitative study explores mealtime care for people with dementia, from the perspective of NH staff. Semi-structured interviews with NH staff (n=16) were carried out in two nursing homes. The homes were chosen to have diverse characteristics: one home had a large number of beds and was part of a small local organization; the other had a small number of beds and was part of a large national organization. Various staff members were interviewed, including direct care staff, senior carers, nurses, managers, and kitchen staff. Interviews were audio-recorded and transcribed verbatim. A constant comparison approach was taken, so that data from early interviews were explored in more depth subsequently. From the analysis, five themes emerged as important in mealtime care for people with dementia living in nursing homes: Setting the right tone; Working well as a team; Knowing the residents; Promoting autonomy and independence; Gently persevering. This work forms part of a larger ethnographic study on the topic, which includes data from residents with dementia, and family carers. Results will inform the development of a staff training intervention to optimize mealtime care for this population.

